# The role of non-canonical Hippo pathway in regulating immune homeostasis

**DOI:** 10.1186/s40001-023-01484-x

**Published:** 2023-11-08

**Authors:** Dagang Tang, Huan Xu, Xing Du

**Affiliations:** 1 Department of Orthopedics, Chongqing Traditional Chinese Medicine Hospital, Chongqing, 400021 China; 2 Department of Ophtalmology, Daping Hospital, Army Medical University, Chongqing, 400012 China; 3 Department of Orthopedics, The First Affiliated Hospital of Chongqing Medical University, No.1 YouYi Road, Yuanjiagang, Yu Zhong District, Chongqing, 400016 China; 4 Orthopedic Laboratory of Chongqing Medical University, Chongqing, 400016 China

**Keywords:** Hippo pathway, Immune homeostasis, Macrophages, Oxidative stress, T-cell differentiation

## Abstract

The Hippo pathway is a crucial signaling pathway that is highly conserved throughout evolution for the regulation of organ size and maintenance of tissue homeostasis. Initial studies have primarily focused on the canonical Hippo pathway, which governs organ development, tissue regeneration, and tumorigenesis. In recent years, extensive research has revealed that the non-canonical Hippo pathway, centered around Mst1/2 as its core molecule, plays a pivotal role in immune response and function by synergistically interacting with other signal transduction pathways. Consequently, the non-canonical Hippo pathway assumes significant importance in maintaining immune system homeostasis. This review concentrates on the research progress of the non-canonical Hippo pathway in regulating innate immune cell anti-infection responses, maintaining redox homeostasis, responding to microenvironmental stiffness, and T-cell differentiation.

## Introduction

Organisms must maintain homeostasis in their internal environment to carry out normal physiological activities. The Hippo pathway was initially discovered in Drosophila melanogaster and was highly conserved throughout evolution, playing a crucial role in regulating organ size and preserving tissue homeostasis [1–3]. In mammals, the core components of the Hippo pathway consist of MST1/2 kinases (Mammalian Ste20-like kinases), WW45 scaffold protein (Salvador homolog SAV1), Lats1/2 kinases (large tumor suppressor 1/2), MOB1 scaffold protein (MOB kinase activator 1), as well as downstream transcriptional coactivators YAP (Yes-associated protein) and TAZ (translational coactivator with PDZ binding motif).

**Fig. 1:**
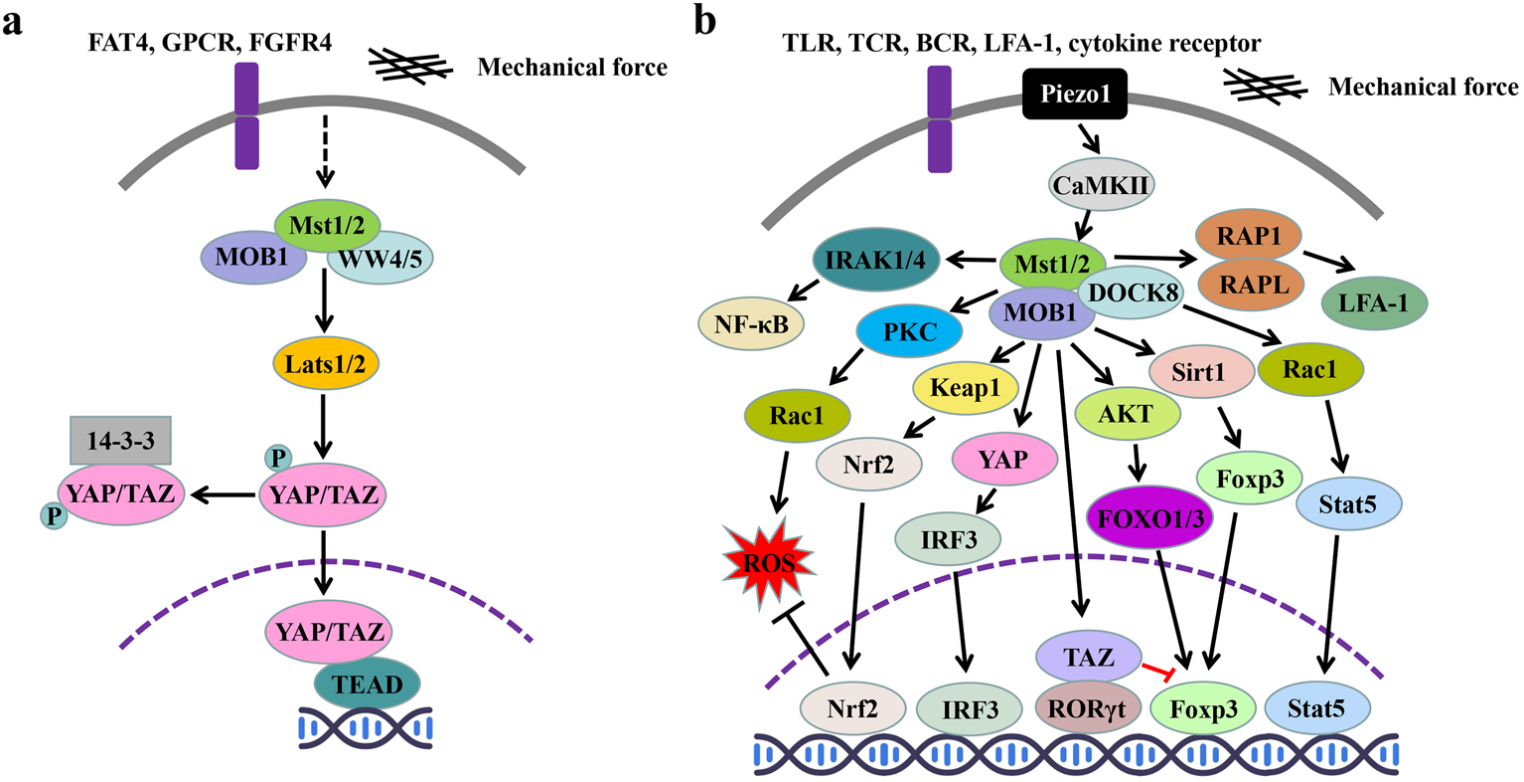
Canonical **a** and non-canonical **b** Hippo signaling pathways. FAT4, atypical cadherin 4; GPCR, G protein coupled receptor; FGFR4, fibroblast growth factor receptor 4; TLR, Toll-like receptor; TCR, T-cell receptor; BCR, B cell receptor; CaMK II, calmodulin-dependent protein kinase II; IRAK1/4, interleukin-1 receptor-related kinase 1/4; NF-κB, nuclear factor κB; PKC, protein kinase C; RAP1, Ras protein 1; RAPL, Ras protein ligand; Rassf5b, Ras-related domain family 5b; LFA-1, lymphocyte function-related antigen 1; DOCK8, cytokinesis factor 8; Rac1, Rac family guanosine triphosphate 1; Keap1, Kelch class ECH-related protein l; AKT, protein kinase B; Sirt1, Sirtuin1; Stat5, signal converting and transcriptional activating protein 5; ROS, reactive oxygen; Nrf2, erythrocyte-like-related factor 2; IRF3, interferon regulatory factor 3; Foxo1/3, fork head box protein O1/3; Foxp3, fork head box protein P3; RORγt, retinoic acid-related orphan receptor γt

In the canonical Hippo pathway, activated MST1/2-WW45 complex phosphorylates and activates the LATS1/2-MOB1A/B complex, activated LATS1/2-MOB1A/B complex then phosphorylates and inactivates YAP and TAZ, leading to YAP/TAZ cytoplasmic retention and degradation. While in nucleus, YAP/TAZ interact with the transcription factor TEAD (translationally enhanced-associated domain) to initiate downstream gene transcription **(**Fig. 1a**).** Numerous studies have demonstrated that the Hippo pathway plays a crucial role in tissue homeostasis, regeneration, and tumorigenesis [4–11]. For example, in mouse liver tissue, inactivation of the Hippo pathway (knockout of the Mst1/2 gene or overexpression of the YAP gene) leads to liver enlargement and eventual hepatocarcinogenesis [10], whereas knockout of the YAP gene leads to hepatocyte death and liver failure [11]. Briefly, the canonical Hippo pathway primarily governs cellular proliferation and death by suppressing the activity of YAP, thereby ensuring tissue and organ homeostasis. Recent studies have increasingly highlighted the pivotal role of the Hippo pathway in maintaining immune system homeostasis.

## Hippo pathway and immune homeostasis

The immune system plays a crucial role in recognizing and eliminating foreign entities, serving as a vital defense mechanism for maintaining internal homeostasis. Under normal physiological conditions, the proper maintenance of immune system homeostasis is essential for its optimal response and functionality. Through diverse immune cells, including those of the adaptive immune system, the immune system continuously monitors the external environment, distinguishing self from non-self, thereby safeguarding against infections, autoimmune disorders, and malignancies [12, 13]. Upon exposure to foreign antigens or autologous cell mutations, the body initiates an immune response aimed at restoring internal homeostasis through a series of appropriate reactions [13, 14]. However, when this response is either too weak or too strong, it can disrupt homeostasis and lead to disease. Immune cells are equipped with receptors that enable them to sense their external environment and trigger corresponding signal transduction pathways which produce downstream effectors. Through possible feedback regulation mechanisms, these responses help maintain bodily homeostasis. Investigating the molecular mechanisms underlying immune homeostasis under both physiological and pathological conditions will enhance our understanding of autoimmune diseases, infections, metabolic disorders and tumors while providing novel ideas and molecular targets for preventing and treating such ailments.

It has been demonstrated that Hippo signaling is essential for maintaining the immune system homeostasis [15]. Unlike the canonical Mst–Lats–Yap signal pathway, which controls tissue growth during development and regeneration, most studies regarding Hippo signaling in immune regulation is focusing in Mst1/2, the core kinases of Hippo signaling, cross-talking with other signaling pathways in various immune cells [15] **(**Fig. 1b**)**.

Early studies have demonstrated the essential role of the Mst1–Nore1b complex (also known as RAPL or Rassf5b) within the Hippo pathway in facilitating LFA-1-mediated T-cell adhesion and migration subsequent to T-cell receptor (TCR) or chemokine activation [16, 17]. The kinase Mst1/2, its binding partner Nore1b, and its substrate MOB1 exhibit high expression levels in immune tissues and cells [8]. Notably, embryonic lethality is observed in Mst1 and Mst2 double gene knockout mice, while single gene knockout of Mst1 primarily manifests as immunodeficiency syndrome. Conversely, no apparent immunodeficiency phenotype is observed in single gene knockout of Mst2. These findings suggest that Mst1 exerts a dominant role within the immune system, with certain complementary functions shared by both Mst1 and Mst2 [8, 18]. A recent study has revealed that the knockdown of MST leads to an enhanced phagocytic activity and improved bacterial clearance in C. hongkongensis hemocytes, highlighting the crucial role of the Hippo pathway in maintaining immune homeostasis and coordinating hemocytic functions [19]. Furthermore, this investigation highlights the divergence between marine invertebrate immunity and mammalian observations regarding the roles of the Hippo pathway, emphasizing the necessity for further comparative studies across different species [19].

Clinical studies have also demonstrated that patients harboring deletion mutations in the kinase MST1 exhibit immunodeficiency syndrome, rendering them susceptible to bacterial, viral, and fungal infections as well as autoimmune diseases. These individuals manifest clinical symptoms such as reduced T cells and B cells within their bodies along with aberrant innate immune cell function [20, 21]. For instance, it has been established that a mutation in MST1 leading to augmented T-cell death during the initial and proliferative phases via the MST1/FOXO1 pathway represents a novel mechanism underlying autosomal recessive primary immunodeficiency [22]. Furthermore, deficiency of STK4 (serine threonine kinase 4), formerly known as MST1, can give rise to an innovative human primary immunodeficiency syndrome characterized by heightened loss of mitochondrial membrane potential and increased susceptibility of lymphocytes and neutrophils to apoptosis [23].

The observed phenotypes and symptoms in mice and patients suggest a crucial role of the Hippo pathway in regulating both innate immunity and acquired immune function. Mst1 deficiency not only leads to anti-infective immune deficiency but also disrupts immune tolerance, resulting in autoimmune diseases. Furthermore, it indicates that the Hippo pathway may exhibit diverse regulatory roles across different immune cells and under varying immune stress conditions. Subsequent investigations have revealed that within the immune system, kinase Mst1/2 primarily modulates the body's immune response through interactions with other signaling pathways involved in regulating immune function. These include integrin signal [24, 25], TCR and B-cell receptor (BCR) signal [26–28], cytokine receptor signal [29, 30], TLR (Toll-like receptor) signal [31–35], mitogen activated protein kinase (MAPK) signal [36, 37], and antiviral signal [38–40] pathways collectively referred to as non-canonical Hippo pathways.

In recent years, significant advancements have been made in the research of the Hippo pathway's role in regulating immune system function and maintaining homeostasis. Initial investigations revealed that deletion of Mst1 resulted in lymphocytopenia. Subsequent studies demonstrated that Mst1 acts as an intrinsic inhibitor of T-cell proliferation upon antigen stimulation or anti-CD3 antibody activation, playing a crucial role in preserving T-cell numbers within the body [18, 28]. Recent findings have also highlighted the ability of Mst1/2 and its downstream molecule TAZ to suppress helper T-cell 17 (Th17) differentiation while promoting regulatory T-cell (Treg) differentiation within T cells. Consequently, precise regulation of the Hippo pathway is indispensable for preventing autoimmune diseases and sustaining immune tolerance specifically within T cells [41]. In addition, studies have revealed that the mechanical receptor Piezo1 interacts with the TLR signal in innate immune cells to activate kinase Mst1/2. This activation promotes ROS production by regulating downstream small G protein Rac1 and modulates phagocytosis as well as the bactericidal effect of phagocytes [32, 33]. Simultaneously, kinase Mst1/2 can also sense cellular ROS levels and regulate antioxidant activity through the Mst1/2-Keap1–Nrf2 signaling pathway, thereby maintaining cellular redox homeostasis [42].

## Hippo signaling and T cells

After migrating to the thymus, lymphoid progenitor cells in the bone marrow undergo a series of selection processes. These include differentiation into CD4^−^CD8^−^ double-negative (DN) thymocytes, further maturation into CD4^+^CD8^+^ double positive (DP) thymocytes, subsequent development into either CD4^+^ or CD8^+^ single-positive (SP) thymocytes, and finally reaching maturity as T cells. Once matured, T cells enter the bloodstream and migrate to secondary lymphoid tissues, where they receive antigen stimulation and activation. T cells that have not yet encountered antigens are referred to as initial or native T cells. They circulate and migrate repeatedly through lymphatic vessels, peripheral blood, and tissue fluid to be widely exposed to potential antigens. This process is crucial for enhancing immune response and maintaining long-term immune memory. Numerous studies have demonstrated the significant role of Mst1 in T-cell migration, homing, differentiation, and activation [25, 28, 30, 41, 43, 44].

### Mst1/2 regulates the activation and migration of T cells

In the thymus, the expression of Mst1 and Mst2 proteins was initially low at the DP stage but gradually increased during development, reaching high levels in CD4^+^ and CD8^+^ SP, MOB1 expression, a substrate of Mst1 and Mst2, remained similar in both DP and SP thymocytes; however, phosphorylated MOB1 was only detected in SP thymocytes. These findings indicate that Mst1 and Mst2 have minimal impact on early thymocyte development (prior to the DP stage) [28]. In peripheral blood, recently matured T cells that migrated from the thymus exhibited high levels of Mst1 and Mst2 expression. Compared to CD4 + CD62L^high^ initial T cells, protein and mRNA levels of Mst1 and Mst2 decreased by approximately 90% in CD4 + CD62L^low^ cells [28]. In addition, several studies have demonstrated that Mst1 knockout mice exhibit heightened T-cell activation and an increased proportion of CD4^+^CD62L^low^ effect/memory T cells. Thus, it can be inferred that the reduction in Mst1 and Mst2 protein levels is crucial for the transition of naive T cells into active effector/memory T cells, with the activity level of Mst1 in T cells determining the threshold for initial T-cell activation. Unlike Mst1, single gene knockout of Mst2 does not impact the number of T cells in various tissues; however, further conditional knockout of Mst2 in hematopoietic cell lines with Mst1 deletion will exacerbate the phenotype of T-cell depletion. This suggests kinase Mst2 plays a compensatory role within lymphoid tissue in response to loss of function mutations in kinase Mst1. Moreover, the activity of kinase Mst1 is essential for maintaining both the quantity and functional stability of T cells, since only transgenic mice expressing wild type rather than kinase-defective forms of Mst1 can restore normal functioning when faced with a deficiency caused by Mst1 deletion [18].

Compared to wild-type mice, Mst1^−/−^Mst2^fl/fl^ Vav–Cre mice (with Mst1 knocked out in the whole body and Mst2 conditionally knocked out in the hematopoietic system) exhibit an increased percentage of CD4^+^ and CD8^+^ single-positive (SP) thymocytes. These SP thymocytes undergo a significant number of apoptotic events and, despite being mature, are unable to migrate out of the thymus [18]. Qa lymphocyte antigen 2 (Qa-2) expression is high during late-stage thymocyte development and continues to increase as mature thymocytes leave the thymus and become peripheral mature T cells. Conversely, CD24 is highly expressed during thymocyte development but decreases when mature thymocytes exit the thymus and is not expressed in peripheral mature T cells [45]. Therefore, Qa-2 and CD24 can serve as surface markers for both thymocytes and peripheral T cells. The majority of CD4^+^ and CD8^+^ SP thymocytes in Mst1^−/−^Mst2^fl/fl^ Vav–Cre mice display a Qa-2^high^CD24^low^ phenotype. In addition, high expression levels of sphingosine 1-phosphate receptor (S1P), primarily observed during late-stage development of thymocytes, indicate that these cells are detected as mature T cells within the thymus [18]. The expression levels of chemokine receptors (such as CCR7, CXCR3, CXCR4 and CCR5) and integrins (CD11b and LFA-1) on the surface of SP thymocytes with Mst1/2 deletion are similar to those of wild-type cells; however, if these cells are placed in the peripheral circulation, they cannot migrate into the secondary lymphoid tissue; it shows that the thymocytes with Mst1/2 deletion can develop and mature into initial T cells, but these cells cannot migrate out of thymus tissue; further study found that in mature thymocytes, under the stimulation of chemokines CCL19 or S1P, Mst1/2 promotes the interaction between MOB1 and DOCK8 by phosphorylating MOB1, enhances the activity of its guanosine conversion factor (GEF), and promotes the activation of Rho family members of small G proteins (such as Rac1), thereby promoting the migration of thymocytes out of the thymus [18].

In Mst1^−/−^Mst2^fl/fl^ Vav–Cre mice and Mst1^−/−^Mst2^fl/fl^ Lck–Cre mice (with Mst1 knocked out in the whole body and Mst2 conditionally knocked out during thymic development), mature thymocytes cannot migrate out of the thymus normally, and peripheral T cells are reduced and highly activated [18]. To further study the function of Mst1/2 in peripheral T cells, Geng J et al. constructed Mst1^fl/fl^Mst2^fl/fl^ Ox40–Cre mice that only knocked out Mst1 and Mst2 in mature and activated T cells. They found that the thymocytes of these mice developed normally, and the number of T cells in peripheral tissues was also normal, but compared with wild-type mice, the proportion of effector/memory T cells increased in the spleen, indicating that Mst1/2 deletion promoted T-cell activation [41].

Upon TCR stimulation, Mst1-deficient initial T cells showed significantly increased proliferation levels and cytokine levels of interleukin-2 (IL-2), IL-4, and interferon γ (IFN-γ), compared to wild-type T cells. However, the phosphorylation levels of downstream TCR molecules, including CD3, ZAP70, Lck, and PLCγ1, remained similar to those of wild-type T cells [28]. Notably, in Mst1-deficient T cells, the phosphorylation level of Lats1/2 remained unchanged, whereas the phosphorylation of MOB1 was significantly diminished. This observation suggests that MOB1 may play a crucial role in T-cell activation [28]. Nevertheless, some studies have suggested that YAP knockout cannot alleviate the defective phenotype caused by Mst1 deletion, thus indicating that Mst1 does not play a significant role in the canonical Hippo pathway in T cells [18, 46]. In addition, under stimulation, the apoptosis ratio of the effect/memory CD4^+^ T cells with Mst1 deletion significantly increased, pointing to a crucial anti-apoptotic role for Mst1 in these T cells. Mst1 deletion, on the other hand, can enhance activation-induced cell death (AICD) effectiveness [28]. Taken together, these results suggest the critical regulatory role of Mst1/2 in maintaining T-cell numbers, migration, and activation.

### Mst1/2-TAZ regulates the differentiation fate of CD4^+^ T cells

Under the regulation of various factors, including TCR/antigen stimulation and cytokines, initial CD4^+^ T cells undergo differentiation to various subtypes of T cells, each performing distinct roles in immune response and regulation [47]. Upon TCR/antigen stimulation, CD4^+^ T cells may differentiate into Treg cells in response to transforming growth factor β (TGF-β) induction. Treg cells are known for secreting TGF-β and for their crucial role in immune tolerance and immunosuppression. Conversely, when co-induced with TGF-β and IL-6, CD4^+^ T cells differentiate into CD4^+^Th17 cells, a cell type known for secreting IL-6 and IL-17 while playing a significant role in defending against extracellular bacterial and fungal infections, inflammatory responses, and autoimmune reactions. The Treg to Th17 cells ratio plays a pivotal role in immune homeostasis. Aberrant differentiation of primary CD4^+^ T cells, displaying either excessively high or low levels of Th17 cells or Treg cells, is often associated with various autoimmune diseases.

The differentiation of Treg and Th17 subtypes is reciprocally regulated, and TGF-β plays a pivotal role in regulating the differentiation of CD4^+^ initial T cells into Treg and Th17 cells. The co-treatment of TGF-β and IL-6 inhibits Treg differentiation and promotes Th17 differentiation, facilitating the body's transition from immune tolerance to immune inflammatory response upon encountering external interferences, such as infections [47]. In 6–8-week-old Mst1^−/−^ mice, the number of CD4^+^ Tregs in the thymus and spleen is significantly reduced, and their regulatory function is also impaired when compared to wild-type mice [44, 48]. Further investigations revealed that Mst1 promotes the expression and function of the transcription factor Foxp3 in Treg cells through various pathways, including direct phosphorylation of Foxo1/3, kinase AKT [44], deacetylases Sirt1 [48], or regulation of the Rac1–DOCK8 pathway [30].

A recent study revealed that Mst1^−/−^ mice exhibited a significantly increased Th17 differentiation ratio and were susceptible to Sjögren’s syndrome and colitis [41]. When mice were exposed to keyhole hemocyanin (KLH) antigen and Freund's complete adjuvant (CFA), a significant increase in the Th17 ratio was observed in the draining lymph nodes of Mst1^fl/fl^Mst2^fl/fl^ Ox40-Cre mice compared to wild-type mice. In addition, the Treg ratio was markedly reduced. Major members of the Hippo pathway were analyzed in various CD4^+^ T-cell subtypes, with significantly elevated levels of transcriptional coactivator TAZ, a downstream kinase of Mst1/2, observed in Th17 and Treg cells compared to initial CD4^+^ T cells.

Moreover, TAZ expression levels were found to be higher in memory T cells in the peripheral blood of rheumatoid arthritis patients and positively correlated with the expression of the core transcription factor ROR of Th17. Mechanistic studies showed that TAZ mRNA expression was significantly increased upon TCR stimulation of initial CD4^+^ T cells in the presence of TGF-β, while IL-6 played a role in enhancing TGF-β-induced TAZ mRNA expression through regulation of transcriptional factors Smad3 and Stat3, respectively.

**Fig. 2:**
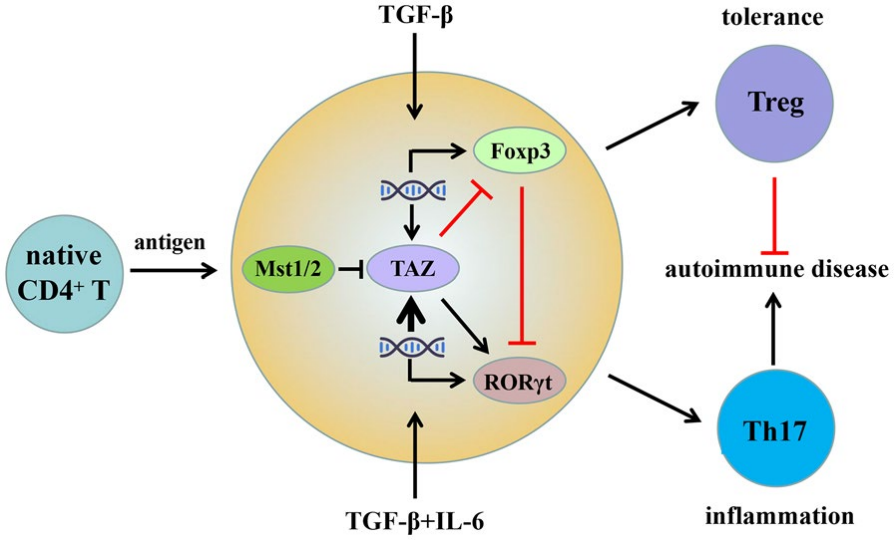
Mst1/2-TAZ signal regulates the differentiation of Th17 and Treg. During the differentiation of Th17 and Treg cells, TGF-β induces the expression, and IL-6 further increases the expression. As a transcription coactivator, TAZ can enhance the core transcription factor RORγt in Th17 cells to promote their development and differentiation of Th17. TAZ can also promote the degradation of Foxp3 and inhibit the differentiation of Tregs by regulating the acetylation and ubiquitination levels of Foxp3, the core transcription factor of Tregs. Therefore, the Hippo pathway regulates homeostasis of immune inflammation and tolerance

Research has also indicated that TAZ can interact and form a complex with the core transcription factors RORγt and Foxp3 in Th17 and Treg [41]. As a transcriptional coactivator, TAZ can directly boost the transcriptional activity of RORγ while also competitively binding to Foxp3 and suppressing acetyltransferase Tip60 (HIV–1TAT interactive protein, 60ku), resulting in weakened differentiation of Treg and promotion of Th17 differentiation [49]. Thus, the Mst1/2-TAZ signaling pathway plays a crucial role in regulating the differentiation of initial CD4^+^ T cells into Th17 and Treg **(**Fig. 2**)**, underlining the importance of the non-canonical Hippo pathway in immune system homeostasis [41].

## Function of Hippo pathway in innate immune cells

The innate immune system serves as a crucial defense mechanism for the body to distinguish self from non-self, promptly respond to external environmental intrusion (such as pathogenic infections), and initiate an immune response in the first instance. Mononuclear macrophages are the most important innate immune cells that trigger inflammatory reactions. They, along with other important innate immune cells, such as neutrophils, play a pivotal role in phagocytosis and pathogen elimination. When pathogens invade the body, innate immune cells recognize antigen-related model molecules expressed in pathogens, such as lipopolysaccharide (LPS), lipoprotein, polypeptide, and nucleic acid molecules, through pattern recognition receptors, such as TLR, C-type lectin-like receptors, scavenger receptors, and complement receptors [50].

TLR is ubiquitously expressed in innate immune cells, enabling recognition of diverse pathogen-associated molecular patterns and serving as the primary line of defense against pathogenic invasion. Moreover, TLR acts as a crucial interlink between the innate and acquired immune responses [12]. A previous study utilized different TLR ligands to activate bone-marrow-derived macrophages (BMDM) derived from wild-type mice. It was found that stimulation and activation of TLR1, TLR2, and TLR4 on the cell surface by corresponding ligands or agonists significantly increased the phosphorylation level of MOB1, a substrate of Mst1/2. However, stimulation of TLR7, TLR8, and TLR9 in endosomes by their respective ligands did not alter MOB1 phosphorylation. Nonetheless, TLR3 expressed on endosomes could mildly elevate MOB1 phosphorylation under poly (I:C) stimulation [33]. The activation of Mst1/2 by LPS (TLR4 agonist), Pam3CSK4 (TLR1 and TLR2 agonists), and LTA (TLR2 agonists) was found to be dependent on MyD88 ((myeloid differentiation primary response gene 88)) signaling via the use of a MyD88-deleted mononuclear macrophage line, RAW264.7. However, Mst1/2 deletion did not affect TLR-mediated MAPK activation (including p38, JNK, and ERK). On the other hand, BMDMs with Mst1/2 deletion showed higher IKKα/β (IκB kinase α/β) and IκBα (inhibitor of NFκB kinase) phosphorylation levels and greater expression of IL-6 and tumor necrosis factor α (TNFα) upon LPS stimulation compared to wild-type BMDMs [33].

In addition, Mst1 regulates the TLR pathway by modulating IRAK1 phosphorylation and, thereby suppressing chronic inflammatory reactions and liver cancer development [34]. Similarly, Mycobacterium tuberculosis also modulates Mst1/2 activity through the TLR2–IRAK1/4 to regulate the expression of chemokine CXCL1/2 and IRF3-mediated response [31]. However, in Dophila melanogaster, studies have that the Hippo signaling is indispensable for resisting gram-positive bacterial and fungal infections. Deletion of the Hippo signal in the Drosophila fat body (the immune organ of Drosophila melanogaster) exhibits a similar immunophenotype Toll receptor signal deletion, suggesting that the canonical Hipp signaling pathway plays a crucial role in regulating innate immune function. When the Hippo signal is inactivated, the downstream transcription coactivator Yorkie is activated, which can directly promote the transcription and expression of Cactus (NFκB inhibitory protein IκB in Drosophila melanogaster), inhibit the nuclear entry of Dorsal (NFκB in Drosophila melanogaster) and dorsal-related immune molecules (Dif), and inhibit the expression of antimicrobial peptide [35]. Studies on mammals have revealed that under the influence of LPS and IFNγ, YAP expression in macrophages is increased, promoting M1-type polarization and IL-6 proinflammatory factor expression. Conversely, stimulation by IL-4 and IL-13 inhibits YAP expression. Therefore, specific knockout of YAP in macrophages can significantly reduce the occurrence of dextran sulfate-induced enteritis [51]. These results indicate that the Hippo pathway is evolutionarily conserved, but that there are more complex regulatory mechanisms that regulate the innate immune response in higher mammals.

### Mst1/2 regulates the function of phagocytes

Patients with Mst1 deficiency are susceptible to various infectious diseases caused by bacteria, viruses, and fungi [20, 21]. Mst1^−/−^Mst2^fl/fl^ Vav–Cre mice often contract pneumonia, lung abscess, and multiple infections. In contrast to Mst1^−/−^Mst2^fl/fl^ Vav–Cre mice, Mst1^fl/fl^Mst2^fl/fl^ Lyz2–Cre mice (with Mst1 and Mst2 conditionally knocked out in myeloid cells) that were raised under standard SPF (specific pathogen-free) conditions for 7 months did not exhibit inflammation or infection. However, compared to wild-type mice, the researchers found that the model mice established by cecal puncture-induced bacterial peritonitis (CLP) in Mst1^fl/fl^Mst2^fl/fl^ Lyz2–Cre mice had a higher mortality rate, greater bacterial loads in various tissues, and higher levels of inflammatory factors [33]. Further study demonstrated that the activation of Mst1/2, which is mediated by TLR–MyD88 signal, is necessary for the phagocytic cells (such as macrophages and neutrophils) to phagocytize and kill bacteria [33]. In the case of Escherichia coli or Listeria monocytogenes infections, the number of bacteria engulfed by BMDM or neutrophils with Mst1/2 deletion is lower than in wild-type cells, and the number of bacteria retained in the cells at the later stage of infection is significantly higher. This suggests that Mst1/2 deletion affects the phagocytosis and killing effectiveness of phagocytic cells on bacteria.

Reactive oxygen species (ROS) play a crucial role in the bactericidal activity of phagocytes. These ROS are mainly produced by the reduced coenzyme II (NADPH) oxidase complex in phagosomes, and mitochondrial ROS (mROS) are produced by the recruited mitochondria. In phagocytes with Mst1/2 deletion, the production of ROS and mROS induced by bacterial infection is markedly deficient, which leads to a decline in bacterial killing efficiency. TLR1, TLR2, and TLR4 activation on the cell membrane can stimulate the generation of significant amounts of mROS and total intracellular ROS, but such activation of TLR3, TLR7, TLR8, and TLR9 expressed on the endosome cannot trigger ROS production. Notably, the stimulation of BMDMs or neutrophils depleted of Mst1/2 with agonists specific to TLR1, TLR2, and TLR4 does not induce mROS or total intracellular ROS production, which was consistent with the results of previous studies that stimulation of these TLRs requires Mst1/2 activation on the membrane surface [33].

In terms of mechanism, phosphorylation of Mst1/2 leads to the activation of PKC, which in turn phosphorylates its substrate LyGDI, a lymphocyte-specific guanine nucleotide dissociation inhibitor. This activation activates small G protein Rac1, facilitates the formation of the TRAF6–ECSIT (TNF receptor-associated factor 6-evolutionarily conserved signaling intermediate in Toll pathway) complex, and subsequently promotes the recruitment of mitochondria in proximity to the phagocyte, releasing mROS to eliminate bacteria within the phagosome. Rac1 is one of the main members of the NADPH oxidase complex, and activated Rac1 promotes the production of phagocytic ROS, further enhancing the ROS level in phagosomes to kill bacteria [33]. This study reveals a novel signal transduction pathway, the TLR–Mst1/2-PKC–Rac1–TRAF6–ECIST mediated mechanism of bacterial-induced ROS production in phagocytes [33]. This indicates that the downstream TLR signal not only activates relevant transcription factors and promotes the expression of inflammatory genes but can also initiate small G proteins through post-translational modification and affect the cytoskeleton to rapidly regulate the migration of organelles, such as phagosomes and mitochondria, thus manifesting an innate immune response against infection.

### Mst1/2 regulates the anti-infection immunity of macrophage

Infection, inflammation, and tumorigenesis cause alterations in the physical microenvironment of tissues, such as stiffness, structure, and composition of interstitial cells. Recent studies show that these physical and mechanical signals in the cellular microenvironment regulate cell differentiation, proliferation, and migration efficiently [52]. As important innate immune cells, macrophages constantly shuttle in body fluids and tissues to cope with complex microenvironments and perceive changes in the physical microenvironment through internal changes in cell stiffness and elasticity. Notably, previous findings demonstrate that cellular growth density and medium stiffness regulate the activity of the Hippo signaling pathway. At a low cellular growth density or on a medium with high stiffness, the Hippo signaling pathway's activity is impeded, causing molecules such as YAP/TAZ to enter the nucleus and promote cell growth. Thus, YAP/TAZ, the two critical transcriptional effectors governed by mechanical force, enable the translation of physical signals into gene expression [53].

A previous study found that the TLR activation signal can activate Mst1/2, and this activation process depends on the MyD88 molecule; however, the activation mechanism of Mst1/2 by TLR–MyD88 complex remains unclear [33]. A recent study found that the phagocytic and bactericidal functions of macrophages cultured on soft substrates are severely impaired, and mechanical stiffness signals from the external environment are necessary for macrophages to achieve bacterial phagocytosis and killing [32]. The Piezo1 ion channel is a mechanical-sensitive Ca^2+^ channel discovered in recent years, which plays an important role in vascular development, red blood cell volume regulation, and cell migration [54]. It was found that the Piezo1 receptor was highly expressed in a variety of myeloid immune cells, and its expression level would further increase under the stimulation of some TLR ligands, such as bacterial infection or LPS [32]. Compared with wild-type mice, the survival rate of Piezo1^fl/fl^ Lyz2–Cre mice (with innate immune cells and Piezo1 knocked-out in the CLP model) was significantly reduced, the bacterial load in various tissues was significantly increased, and the phenotype of BMDM with Mst1/2 deletion was similar to that of BMDM with Piezo1 deletion; the bacterial phagocytosis and killing ability of BMDM with Piezo1 deletion were significantly lower than those of wild-type BMDM. Treatment with Yoda1 (Piezo1 activator) significantly enhanced the bacterial phagocytosis and killing ability of wild-type BMDM, but had no effect on BMDM with Piezo1 deletion, indicating that activation of Piezo1 can promote the anti-infection ability of macrophages. Bacterial infection or LPS stimulation promotes the assembly of TLR4 and Piezo1, thus enhancing the calcium influx, which is dependent on Piezo1 and the activation of CaMK II. CaMK II then mediates the activation of Rac1 by phosphorylating the Mst1/2 of the Hippo pathway, promoting the remodeling of filamentous actin, enhancing phagocytosis, and promoting the production of ROS in mitochondria and phagosomes, thus enhancing bactericidal ability. These results indicate that the mechanoreceptor Piezo1 is a key upstream molecule that activates the Mst1/2-Rac1 anti-infection signal and induces lethal ROS production [32]. Recently, it has also been found that Piezo1 can regulate macrophages to phagocytosis and clear senescent red blood cells and iron metabolism [55], and sense periodic changes in hydrostatic pressure [56], thus transforming extracellular signals into intracellular signals to regulate the inflammatory responses of cells. These studies revealed the role and mechanism of mechanical force sensing and response signals in the anti-infection of macrophages, indicating that the mechanical force transmission signal mediated by Piezo1 is crucial for the innate immune response of the host against pathogens.

### Mst1/2 regulates redox homeostasis of macrophages

ROS play a vital role in maintaining normal cellular function and are the primary defense mechanism utilized by immune phagocytes to eliminate pathogens and activate inflammation. However, excessive ROS can lead to oxidative stress damage, resulting in cellular aging and death. Thus, regulating ROS production and clearance is essential for maintaining cellular redox homeostasis. In vitro study, hydrogen peroxide solution is often used to activate Mst1/2, indicating that ROS can effectively activate Mst1/2 [28]. Previous research has shown that deletion of CST-1 (the homologous molecule of Hippo kinase) in the nematode (Caenorhabditis elegans) accelerates aging and reduces lifespan [57]. Furthermore, Mst1/2 is crucial for macrophages to induce high ROS levels during bacterial infections, but macrophages lacking Mst1/2 exhibit significantly higher ROS levels than wild-type cells [33].

In macrophages deficient in Mst1/2, levels of protein carbonylation (a marker of oxidative stress), phosphorylation of H2A.X (a marker of DNA damage), PARPγ (poly ADP-ribose polymerase γ) and cleaved-Caspase3 expression were all increased, indicating characteristics of early aging. These phenomena could be alleviated by treatment with the ROS scavenger N-acetylcysteine (NAC), indicating that Mst1/2 plays an important role in regulating macrophage oxidative stress homeostasis [42]. The results of the study showed that Mst1/2 deletion led to defects in the macrophage antioxidant enzyme system. The expression of antioxidant enzyme genes such as Nqo1, Ho-1, Gclc, and Gclm were significantly lower in BMDM with Mst1/2 deletion when compared to wild-type BMDM, following treatment with LPS, antimycin A, rotenone, hydrogen peroxide, or E. coli infection. These antioxidant genes are mostly regulated by the antioxidant transcription factor Nrf2, which is highly expressed in macrophages. Nrf2 ubiquitination levels in macrophages with Mst1/2 deletion were significantly increased, and protein levels were significantly decreased. Further research shows that Mst1/2 senses ROS and is recruited to phagosomes and mitochondria to be activated by released ROS, thereby regulating Nrf2 and maintaining redox homeostasis in cells. Mst1/2 thus protects cells from oxidative damage, delaying aging and death during pathogen clearance by macrophages.

**Fig. 3:**
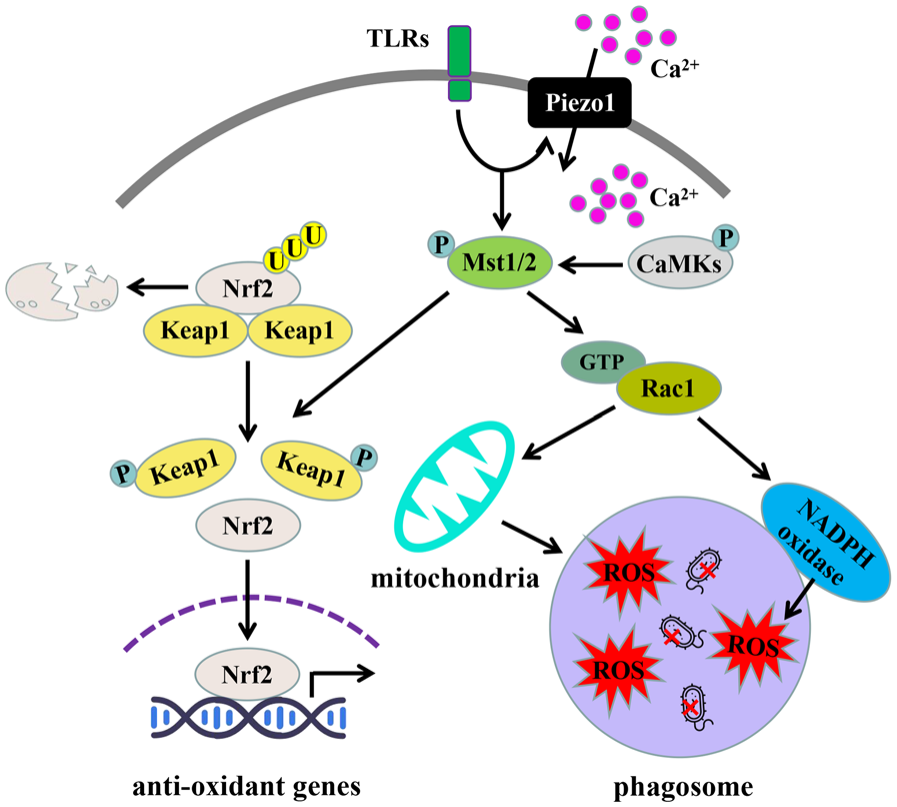
Molecular mechanism of Mst1/2 regulating macrophage anti-infection and maintaining cell redox homeostasis. Under the condition of bacterial infection, TLR in macrophages combined with the mechanical sensitive receptor Piezo1 activates Mst1/2, activates Rac1, promotes mitochondria to collect around phagosomes to produce lethal ROS, and enhances NADPH oxidase activity on phagosome cell membranes to produce more lethal ROS, ultimately killing bacteria. A large amount of ROS further activates Mst1/2, regulates the stability of Nrf2 through phosphorylation of Keap1, promotes the expression of antioxidant genes, and realizes the antioxidant stress response of cells. Therefore, the Hippo pathway plays an important role in regulating redox homeostasis in macrophages

The Nrf2 protein in cells is strictly regulated by the ubiquitin proteasome system. Under normal physiological conditions, the Keap1 and Cul3–E3 ubiquitin ligases target Nrf2, promoting its ubiquitination and subsequent proteasome degradation. However, during oxidative stress, the depolymerization of Keap1 inhibits the binding of Keap1–Cul3–E3 to Nrf2, resulting in decreased Nrf2 ubiquitination and increased protein levels. Nrf2 then enters the nucleus and binds with the ARE sequence, activating downstream antioxidant stress kinase [58, 59]. Regulatory mechanism study has confirmed that Mst1/2 can bind directly to Keap1 and phosphorylate four sites, preventing the polymerization of Keap1 molecules and blocking the degradation of Nrf2. Additional expression of Nrf2 via a viral vector has shown significant reductions in ROS levels, DNA damage, and cell apoptosis in macrophages with Mst1/2 deletion. These findings confirm that Nrf2 is a key downstream molecule of Hippo kinase in macrophages. This research reveals a new antioxidant and anti-aging signaling pathway during the participation of macrophages in host defense, clarifying the key mechanism by which Mst1/2 regulates macrophages in maintaining oxidative stress homeostasis while avoiding ROS-induced self-injury and cell aging **(**Fig. 3**)**. This provides an important theoretical basis for further research on how oxidative stress promotes the occurrence and development of aging-related inflammation and infection.

## Summary and prospect

The Hippo pathway is conserved throughout evolution and plays a critical role in regulating cell proliferation, apoptosis, and tissue/organ homeostasis, making it a major tumor suppression pathway. Recent studies have established the Hippo pathway as a key regulator of immune homeostasis. In macrophages, the core kinase Mst1/2 acts as a molecular switch controlling both the signal to induce lethal ROS and the signal to activate Nrf2 to clear excess ROS, thus maintaining redox homeostasis [32, 33, 42]. These findings highlight the importance of coordinated intracellular communication to ensure proper immune responses to various external stimuli. Defects in regulatory links can impair phagocytosis and pathogen clearance, damage redox balance, and even lead to premature cell aging and death.

In terms of acquired immune regulation, the Hippo pathway inhibits the differentiation of pro-inflammatory Th17 cells and promotes the differentiation of immunosuppressive Tregs, which are crucial for maintaining tolerance and stability of the immune system under normal physiological conditions. This elucidates the pathological mechanism of patients susceptible to autoimmune diseases when there is an imbalance in the Hippo signaling pathway. These findings suggest that the non-canonical Hippo pathway may serve as a potential therapeutic target for the management of infectious and autoimmune diseases, immune cell aging, tumors, and other immune disorders.

In targeting and promoting the Hippo pathway for tumor therapy, consideration must be given to the role of the pathway in promoting Treg cells differentiation and function, which can hinder immune system elimination of tumor cells. However, this approach may have potential benefits in treating autoimmune diseases by inhibiting Th17 differentiation and enhancing the anti-inflammatory effects of Tregs cells. The Hippo pathway plays a multifaceted role in regulating tumor development, tissue homeostasis, and immune response. Directly targeting core node molecules of this pathway could have unintended consequences. Therefore, when developing small-molecule drugs, factors such as site of action, efficiency, and reversibility should be considered. For instance, XMU-MP-1 is a small-molecule drug that specifically and reversibly inhibits kinase Mst1/2, allowing for precise medication to promote regeneration without causing abnormal tissue proliferation. This drug has proven successful in animal models of liver and intestinal regeneration [60].

Furthermore, by investigating the precise regulatory function of the Hippo pathway within the immune system, particularly its cooperation with other commonly recognized immune signaling pathways and its role in regulating interactions between tumors and immune cells, as well as identifying specific effectors in diverse tissue microenvironments and immune response events, we can effectively target the Hippo pathway as a therapy for conditions, such as tumors, injury regeneration, infections, and autoimmune diseases.

## Data Availability

Not applicable.
